# Five-year Estimated Glomerular Filtration Rate in Patients With Hypoparathyroidism Treated With and Without rhPTH(1–84)

**DOI:** 10.1210/clinem/dgaa490

**Published:** 2020-08-01

**Authors:** Kristina S Chen, Elvira O Gosmanova, Gary C Curhan, Markus Ketteler, Mishaela Rubin, Elyse Swallow, Jing Zhao, Jessie Wang, Nicole Sherry, Alan Krasner, John P Bilezikian

**Affiliations:** 1 Shire Human Genetic Therapies, Inc., Cambridge, Massachusetts (a Takeda company); 2 Division of Nephrology, Albany Medical College and Nephrology Section, Stratton VA Medical Center, Albany, New York; 3 Renal Division, Brigham and Women’s Hospital, Harvard Medical School, Boston, Massachusetts; 4 Department of General Internal Medicine and Nephrology, Robert-Bosch-Krankenhaus, Stuttgart, Germany; 5 Department of Medicine Program, University of Split School of Medicine, Split, Croatia; 6 Columbia University College of Physicians and Surgeons, New York, New York; 7 Analysis Group Inc., Boston, Massachusetts

**Keywords:** chronic hypoparathyroidism, kidney function, recombinant human parathyroid hormone (1-84), retrospective study

## Abstract

**Context:**

Chronic hypoparathyroidism (HypoPT) is conventionally managed with oral calcium and active vitamin D. Recombinant human parathyroid hormone (1–84) (rhPTH[1–84]) is a therapy targeting the pathophysiology of HypoPT by replacing parathyroid hormone.

**Objective:**

To compare changes in the estimated glomerular filtration rate (eGFR) in patients with chronic HypoPT receiving or not receiving rhPTH(1–84) during a 5-year period.

**Design/Setting:**

A retrospective analysis of patients with chronic HypoPT treated with or without rhPTH(1–84).

**Patients:**

Sixty-nine patients with chronic HypoPT from 4 open-label, long-term trials (NCT00732615, NCT01268098, NCT01297309, and NCT02910466) composed the rhPTH(1–84) cohort and 53 patients with chronic HypoPT not receiving rhPTH(1–84) from the Geisinger Healthcare Database (01/2004–06/2016) composed the historical control cohort.

**Interventions:**

The rhPTH(1–84) cohort (N = 69) received rhPTH(1–84) therapy; the historical control cohort (N = 53) did not receive rhPTH(1–84).

**Main Outcome Measures:**

Changes in eGFR from baseline during a 5-year follow-up were examined in multivariate regression analyses.

**Results:**

At baseline, demographic characteristics and eGFR were similar between cohorts, though the proportions with diabetes and cardiac disorders were lower in the rhPTH(1–84) cohort. At the end of follow-up, mean eGFR increased by 2.8 mL/min/1.73 m^2^ in the rhPTH(1–84) cohort, while mean eGFR fell by 8.0 mL/min/1.73 m^2^ in the control cohort. In the adjusted model, the difference in the annual eGFR change between the rhPTH(1–84) cohort and the control cohort was 1.7 mL/min/1.73 m^2^ per year (*P* = 0.009).

**Conclusions:**

Estimated glomerular filtration rate was preserved for over 5 years among patients with chronic HypoPT receiving rhPTH(1–84) treatment, contrasting with an eGFR decline among those not receiving rhPTH(1–84).

Hypoparathyroidism (HypoPT) is an endocrine disorder, with a prevalence of 10 to 40 cases per a population of 100 000 in the United States and European countries, in which production of parathyroid hormone (PTH) by the parathyroid gland is absent or inappropriately low (1–6). The most common cause of HypoPT is damage to or removal of the parathyroid glands during neck surgery, although it may also be idiopathic or due to autoimmune or congenital etiologies (7–9).

Typically, HypoPT is associated with hypocalcemia, hyperphosphatemia, and hypercalciuria (10). Parathyroid hormone deficiency causes hypocalcemia due to reduced calcium reabsorption from renal tubules, decreased absorption of calcium from the gastrointestinal tract, and decreased calcium mobilization from bone (1). The symptoms of HypoPT, including fatigue, numbness of the distal extremities, muscle cramps, and neurocognitive deficit can vary from mild to severe (2). More serious and potentially life-threatening effects of hypocalcemia such as seizures, cardiac arrhythmias, cardiomyopathy, and laryngeal spasm occur in severe cases.

Conventional management of HypoPT consists of oral calcium supplementation (eg, calcium carbonate or citrate) and active vitamin D treatments (eg, calcitriol or alfacalcidol). While conventional therapy increases intestinal calcium absorption and serum calcium levels, it does not eliminate the symptoms or alleviate nonsymptomatic complications caused by the absence of PTH on the bones and kidneys. In fact, raising serum calcium and phosphate in the absence of PTH’s calcium-retaining effect in the kidney may exacerbate nephrologic complications like nephrolithiasis, nephrocalcinosis, and potential renal dysfunction (10–15).

Recombinant human parathyroid hormone (1–84) (rhPTH[1–84]) has been used in patients with HypoPT. It was approved in 2015 by the US Food and Drug Administration and conditionally approved by the European Medicines Agency in 2017 as an adjunctive therapy in patients with chronic HypoPT who are not well controlled on conventional therapy alone (13, 16). The efficacy and safety of rhPTH(1–84) in HypoPT were evaluated in the 24-week, randomized, double-blind, placebo-controlled trial known as the REPLACE study and the open-label extension trial known as the RACE study (17). The REPLACE study reported that, compared with placebo, significantly more patients treated with rhPTH(1–84) achieved at least a 50% reduction in vitamin D dose and oral calcium supplementation while maintaining acceptable serum calcium levels (17, 18). However, the trial duration of REPLACE (24 weeks) was not long enough to assess the impact of rhPTH(1–84) as an adjunct therapy to conventional treatment on long-term kidney function. Although the impact of rhPTH(1–84) on the long-term estimated glomerular filtration rate (eGFR) in patients with HypoPT has been assessed in the RACE and NCT02910466 trials, no long-term head-to-head clinical trial has been conducted to compare the longitudinal change in eGFR among patients with chronic HypoPT treated with or without rhPTH(1–84) (18–20).

In this retrospective analysis, we addressed this knowledge gap by comparing the changes in kidney function during a 5-year period, as assessed by changes in eGFR, between patients with chronic HypoPT on long-term treatment with rhPTH(1–84) and a historical control cohort of patients with chronic HypoPT not receiving rhPTH(1–84).

## Methods

### Data source

#### rhPTH(1–84) cohort.

The cohort receiving rhPTH(1–84) consisted of patients from the REPLACE (NCT00732615), RELAY (NCT01268098), RACE (NCT01297309), and NCT02910466 trials (18, 19). Collectively, the REPLACE, RELAY, and RACE studies are sequential phase III, long-term, open-label trials designed to assess the long-term safety and tolerability of rhPTH(1–84) among patients with chronic HypoPT (18). NCT02910466 was a phase IV, long-term, open-label trial evaluating the safety as well as the impact of rhPTH(1–84) on the long-term control of serum calcium and on the quality of life among patients with chronic HypoPT (19).

#### Historical control cohort.

The MedMining Electronic Health Records (EHR) database (January 2004–June 2016) was used to construct the historical control cohort of patients with chronic HypoPT who were not treated with rhPTH(1–84). The MedMining EHR database is owned and maintained by Geisinger Health System, which covers 68 counties and nearly 5 million patients in Pennsylvania, United States. The patients in this system received continuous care for a median of 15 years and had a low out-migration rate (under 4% every 4 years) (21).

### Study design

#### Sample selection.

rhPTH(1–84)-treated patients enrolled in the RACE and NCT02910466 trials with eGFR available at baseline and at 5 (±3 months) years were included in the rhPTH(1–84) cohort. For RACE patients, the baseline visit was the closest date of eGFR measurement prior to or on the date of rhPTH(1–84) initiation in either the REPLACE, RELAY, or RACE trial.

The historical control cohort was selected from the MedMining EHR database using criteria similar to the enrollment criteria for the RACE and NCT02910466 trials (key enrollment criteria are summarized in Table 1). Patients included in the control cohort met all of the following criteria: (1) adults aged 18 years and older at the index date; (2) no use of rhPTH(1–84) or rhPTH(1–34); (3) being diagnosed with HypoPT on 2 distinct days; (4) at least 2 eGFR values on or after the first HypoPT diagnosis, 1 on the index date and the other at 5 years (±3 months) after the index date; (5) at least 18 months between the first HypoPT diagnosis and the index date; (6) serum creatinine at the index date <1.5 mg/dL; (7) the most recent total serum calcium assessed within 6 months prior to the index date was ≤10.6 mg/dL; and (8) no thyroid cancer during the 5 years prior to the index date. HypoPT diagnosis was based on the diagnosis codes published in the International Classification of Diseases, Ninth Revision, Clinical Modification (ICD-9-CM); 252.1 in the ICD-9-CM, and in the ICD-10-CM: E20.0, E20.8, E20.9, and E89.2. Medical history and concomitant medication use of the historical control cohort was identified by diagnosis and Generic Product Identifier (GPI)/Healthcare Common Procedure Coding System (HCPCS) drug codes.

**Table 1.** Key selection criteria of the RACE and NCT02910466 trials

Criteria	RACE (NCT01297309)	NCT02910466
Disease diagnosis	Patients with confirmed chronic HypoPT at screening	Patients with confirmed chronic HypoPT at screening
Serum creatinine at baseline	Patients with serum creatinine at baseline <1.5 mg/dL	Serum creatinine <1.5 mg/dL on a single measurement prior to use of study drug
Serum calcium at baseline	Patients with total serum calcium at baseline ≤10.6 mg/dL	–
Age	Age between 18 and 85 years	Age between 18 and 85 years
Medical history	Patients without history of thyroid cancer during 5 years preceding enrollment	Patients with a history of thyroid cancer must be documented to be disease-free for a period of at least 5 years (or at least 2 years with evidence of follow-up and a doctor’s note of clearance)
Thyroid function	Serum thyroid function tests within normal laboratory limits at screening for all subjects not receiving thyroid hormone replacement therapy	Serum thyroid function tests within normal laboratory limits at screening for all subjects not receiving thyroid hormone replacement therapy

Abbreviation: HypoPT, hypoparathyroidism.

#### Study periods.

The index date was defined as the baseline visit for the rhPTH(1–84) cohort and as the earliest of the potential index dates for the control cohort. For both cohorts, the study period was defined from the index date to 5 years (±3 months) postindex date.

#### Study measures.

Baseline characteristics assessed before or at the index date included demographics (age, sex, race, and weight) and concomitant use of medication with potential kidney function influence at the index date (nonsteroidal anti-inflammatory drugs [NSAIDs], proton-pump inhibitors [PPIs], cimetidine, angiotensin-converting enzyme [ACE] inhibitors, angiotensin II receptor blockers [ARBs], and diuretics). In addition, patients’ medical histories (based on ICD-9-CM diagnostic codes during the preindex period) were assessed for hypocalcemia, hypercalcemia, hypertension, type 2 diabetes mellitus, and cardiac disorders. All cardiac disorders reported as medical history in the REPLACE, RELAY, RACE, or NCT02910466 trial were assessed, which included the following conditions: supraventricular tachycardia, cardiac murmur, chest pain, mitral valve prolapse, congestive heart failure, tachycardia, bradycardia, palpitations, sinus bradycardia, chronotropic incompetence, and ventricular pre-excitation. Baseline serum calcium level and serum creatinine level were recorded. Estimated glomerular filtration rate was calculated using the Chronic Kidney Disease Epidemiology Collaboration (CKD-EPI) equation (22).

#### Study outcomes.

 Study outcomes evaluated during the 5-year (±3 months) follow-up period included: (1) the predicted eGFR change from baseline at each year from Year 1 to Year 5 in the rhPTH(1–84) and historical control cohorts, and (2) the difference in the annual rate of eGFR change between the rhPTH(1–84) and historical control cohorts, defined as the difference in slopes of eGFR change over the 5 years between the rhPTH(1–84) cohort and the control cohort.

### Statistical analysis

#### Description and comparison of baseline characteristics. 

Means and standard deviations (SDs) were reported for continuous variables; frequencies and percentages were reported for categorical variables. Baseline characteristics were compared between the 2 cohorts using 2 sample *t*-tests for normally distributed continuous variables and Fisher’s exact test for categorical variables.

#### Description and comparison of eGFR annual change over the 5-year period.

All eGFR data during the 5-year follow-up period following the index date were used to fit unadjusted and adjusted mixed-effects models. The correlation between repeated measures from the same patient was accounted for using random effects adjustment. The dependent variable was eGFR change from the index date to 5 years post-index. The primary independent variable was the interaction term between the study cohort (with the historical control cohort as the reference group) and time, which represents the difference in the annual rate of eGFR change from baseline between the rhPTH(1–84) and control cohorts.

The adjusted regression model controlled for baseline characteristics, including age, sex, history of hypocalcemia, hypercalcemia, hypertension, type 2 diabetes mellitus, cardiac disorders, use of medications with potential influence on kidney function at the index date, and baseline eGFR. Model parameter estimates, 95% confidence intervals (CIs), and *p*-values of the independent variables were reported for both the unadjusted and adjusted models. A *p*-value of 0.05 was used to determine statistical significance.

The trajectories of eGFR change over 5 years for the 2 cohorts were plotted using the predicted eGFR changes from baseline and its corresponding 95% CI based on the unadjusted model.

Slopes of eGFR change over time in 2 cohorts were compared using both unadjusted and adjusted mixed-effects regression models. For both cohorts, the predicted eGFR change from baseline at each year from Year 1 to Year 5 following the index date was estimated based on the adjusted regression model.

#### Sensitivity analysis: propensity score weighting.

In the sensitivity analysis, propensity score weighting was used to adjust for differences in the baseline characteristics between cohorts. Patients in the rhPTH(1–84) and control cohorts were weighted based on their propensity score. A logistic regression model was used to estimate the propensity score (ie, the probability that a patient was treated with rhPTH[1–84]) for each patient. The independent variables included age, sex, baseline eGFR, history of hypertension, cardiac disorders, hypercalcemia, hypocalcemia, type 2 diabetes mellitus, and prescription use of NSAIDs, PPIs, and cimetidine at the index date. Race and the use of ACE inhibitors, ARBs, and diuretics were not included in the propensity score model because all patients in the historical control cohort were white and none of them used ACE inhibitors, ARBs, or diuretics. Weight was not included in the propensity score model due to missing data. After weighting, the baseline characteristics were summarized and compared between study cohorts.

A comparison of eGFR change over time between the rhPTH(1–84) and control cohorts was conducted in the propensity score weighted population using a similar approach as the main analysis.

## Results

### Baseline characteristics

**Figure 1.**
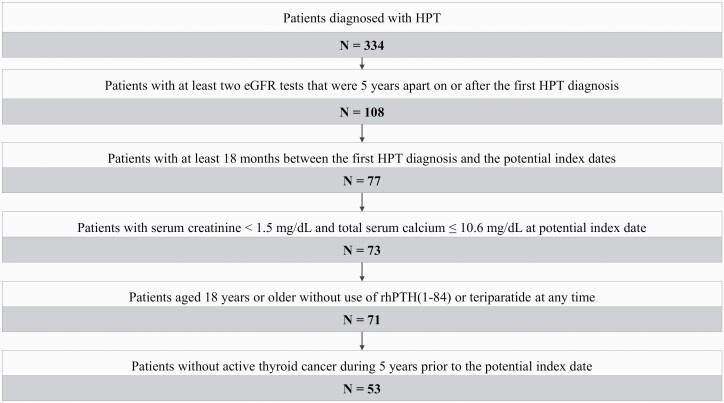
Sample selection of the historical control cohort. Abbreviations: eGFR, estimated glomerular filtration rate; HPT, hypoparathyroidism; rhPTH, recombinant human parathyroid hormone.

A total of 69 patients from the RACE (n = 41) and NCT02910466 (n = 28) trials with eGFR values at the index date and at 5 years (±3 months) were included in the rhPTH(1–84) cohort. A total of 53 patients were included in the historical control cohort (Fig. 1). Baseline characteristics are summarized in Table 2. The demographic characteristics were largely similar between the 2 study cohorts except that the rhPTH(1–84) cohort was younger than the historical control cohort at baseline (48.2 vs 55.8 years, *P *< 0.01). The majority of the patients in both cohorts were white and female with similar weight (Table 2). A significantly lower proportion of patients in the rhPTH(1–84) cohort used NSAIDs, PPIs, and/or cimetidine compared with the control cohort (18.8% vs 50.9%, respectively; *P *< 0.01). Additionally, a significantly lower proportion of patients in the rhPTH(1–84) cohort had a history of hypocalcemia (5.8% vs 41.5%), type 2 diabetes mellitus (2.9% vs 15.1%), and cardiac disorders (15.9% vs 41.5%) compared with the control cohort (all *P *< 0.05). Mean eGFR values at baseline were similar between the study cohorts (rhPTH[1–84] vs control: 77.4 vs 80.5 mL/min/1.73 m^2^, respectively; *P* = 0.42). Serum calcium (rhPTH[1–84] vs control: 8.8 vs 8.7 mg/dL; *P* = 0.99) and serum creatinine levels (1.0 vs 0.9 mg/dL; *P* = 0.11) were comparable between the 2 groups.

**Table 2.** Baseline characteristics

	rhPTH(1–84) Cohort		Historical Control Cohort		*P*-value	
	N = 69		N = 53			
**Demographics**						
Age (years), mean (SD)	48.2	(10.6)	55.8	(18.0)	0.005	*
Female, n (%)	53	(76.8)	39	(73.6)	0.682	
Race, n (%)						
White	67	(97.1)	53	(100.0)	0.996	
Asian	2	(2.9)	0	(0.0)	–	
Weight (kg)^*a*^	91.1	(26.2)	83.0	(22.0)	0.113	
**Concomitant medication use at the index date, n (%)**						
NSAIDs, PPIs, and cimetidine	13	(18.8)	27	(50.9)	<0.001	*
NSAIDs	8	(11.6)	19	(35.8)	<0.001	*
PPIs	6	(8.7)	11	(20.8)	0.064	
Cimetidine	0	(0.0)	0	(0.0)	–	
ACE inhibitors, ARBs, and diuretics	4	(5.8)	0	(0.0)	–	
ACE inhibitors and ARBs	5	(7.2)	0	(0.0)	–	
Diuretics	2	(2.9)	0	(0.0)	–	
**Medical history, n (%)**						
Hypocalcemia	4	(5.8)	22	(41.5)	<0.001	*
Hypercalcemia	0	(0.0)	7	(13.2)	–	
Hypertension	16	(23.2)	21	(39.6)	0.052	
Type 2 diabetes mellitus	2	(2.9)	8	(15.1)	0.028	*
Cardiac disorders^*b*^	11	(15.9)	22	(41.5)	0.002	*
**Lab values**						
eGFR (mL/min/1.73 m^2^), mean (SD)	77.4	(17.2)	80.5	(24.1)	0.416	
Serum calcium (mg/dL)	8.8	(0.9)	8.7	(1.0)	0.990	
Serum creatinine (mg/dL)	1.0	(0.2)	0.9	(0.2)	0.110	

Abbreviations: ACE, angiotensin converting enzyme; ARB, angiotensin II receptor blockers; eGFR, estimated glomerular filtration rate; NSAID, nonsteroidal anti-inflammatory drug; PPI, proton-pump inhibitor; rhPTH, recombinant human parathyroid hormone; SD, standard deviation.

*denotes *P-*value <0.05.

^*a*^Weight at baseline was available for 41 patients in the rhPTH(1–84) cohort and 51 patients in the control cohort, respectively.

^*b*^Cardiac disorders included supraventricular tachycardia, cardiac murmur, chest pain, mitral valve prolapse, congestive heart failure, tachycardia, bradycardia, palpitations, sinus bradycardia, chronotropic incompetence, and ventricular pre-excitation.

### Comparison of 5-year eGFR change between the rhPTH(1–84) and control cohorts

The median (interquartile range) number of eGFR values per patient during the 5-year follow-up was 17 (15–23) and 58 (35–104) for the rhPTH(1–84) and historical control cohorts, respectively. The mean (SD) serum creatinine levels at the end of the 5-year follow-up were 0.9 (0.24) and 1.0 (0.40) mg/dL among the rhPTH(1–84) and historical control cohorts (*P* = 0.07), respectively. The mean (SD) eGFR values among the rhPTH(1–84) and historical control cohorts at the end of 5 years were 79.9 (19.2) and 72.5 (25.8) mL/min/1.73 m^2^ (*P* = 0.07), respectively.

**Figure 2.**
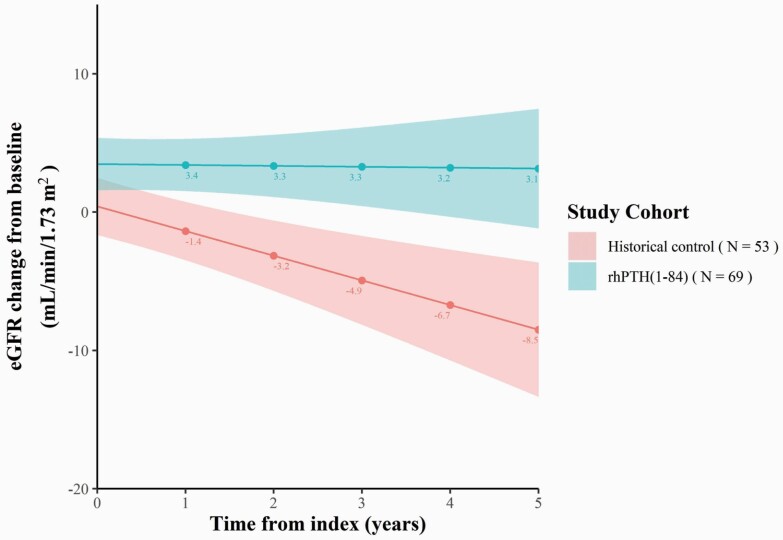
eGFR change from baseline based on the unadjusted regression model.^a^ Abbreviations: eGFR, estimated glomerular filtration rate; rhPTH, recombinant human parathyroid hormone. ^a^ The shaded areas around the lines indicate confidence intervals.

**Figure 3.**
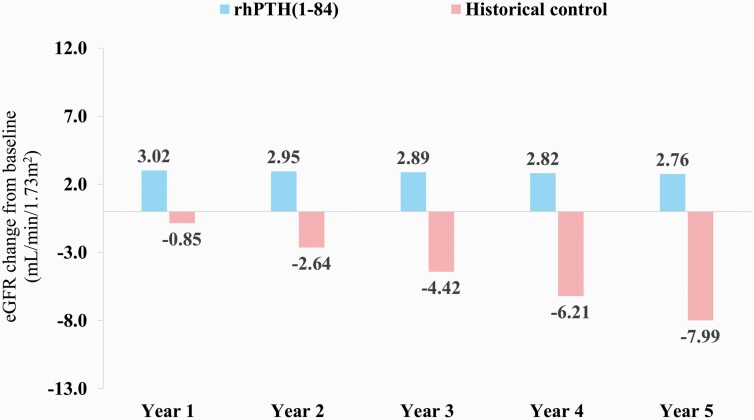
eGFR change from Year 1 to Year 5 based on an adjusted regression model. Abbreviations: eGFR, estimated glomerular filtration rate; rhPTH, recombinant human parathyroid hormone.

In the unadjusted analysis, the slopes of eGFR change were -0.1 mL/min/1.73 m^2^ per year (*P* = 0.88) in the rhPTH(1–84) cohort and -1.8 mL/min/1.73 m^2^ (*P *< 0.01) in the historical control cohort (Fig. 2). The difference in slopes of eGFR change over 5 years between the rhPTH(1–84) and historical control cohorts was 1.7 mL/min/1.73 m^2^ per year (*P* = 0.01), with eGFR remaining higher in the rhPTH(1–84) group at all timepoints. The predicted eGFR changes at baseline were not zero due to the linearity assumption of the models. In the regression model adjusted for age, sex, medical history, concomitant medication use, and baseline eGFR, the difference in the slopes of eGFR change between cohorts was unchanged at 1.7 mL/min/1.73 m^2^ per year (*P *< 0.01). At Year 5, the predicted eGFR based on the adjusted regression model increased by 2.8 mL/min/1.73 m^2^ in the rhPTH(1–84) cohort and declined by 8.0 mL/min/1.73 m^2^ in the historical control cohort (*P *< 0.01) (Fig. 3).

### Sensitivity analysis

Overall, the baseline characteristics were more balanced in the weighted cohorts, with no significant differences in the variables included in the propensity score model (Table 3). In both the unadjusted and the adjusted regression models, the differences in the slopes of eGFR change over the 5 years between the rhPTH(1–84) and control cohorts were estimated at 1.7 mL/min/1.73 m^2^ per year (both *P *< 0.05). Similar to the main analyses, eGFR increased by 3.2 mL/min/1.73 m^2^ in the rhPTH(1–84) cohort and declined by 8.4 mL/min/1.73 m^2^ in the historical control cohort at Year 5 (*P *< 0.01).

**Table 3.** Baseline characteristics (sensitivity analysis)

	rhPTH(1–84) Cohort		Historical Control Cohort		*P*-value	
**Demographics**						
Age (years), mean (SD)	48.8	(10.7)	50.2	(17.5)	0.187	
Female, %		78		78	0.995	
Race, %						
White		97		100	<0.001	*
Asian		3		0	–	
Weight (kg)	90.8	(25.9)	78.0	(17.9)	0.007	*
**Concomitant medication use at the index date, %**						
NSAIDs, PPIs, and cimetidine		27		33	0.598	
NSAIDs		18		24	0.506	
PPIs		11		12	0.338	
Cimetidine		0		0	–	
ACE inhibitors, ARBs, and diuretics		6		0	–	
ACE inhibitors and ARBs		8		0	–	
Diuretics		3		0	–	
**Medical history, %**						
Hypocalcemia		10		20	0.229	
Hypercalcemia		0		6	–	
Hypertension		26		26	0.988	
Type 2 diabetes mellitus		3		7	0.333	
Cardiac disorders		23		26	0.707	
**Lab values, mean (SD)**						
eGFR (mL/min/1.73 m^2^)	78.7	(17.0)	79.2	(22.7)	0.908	
Serum calcium (mg/dL)	8.8	(0.9)	8.5	(1.2)	0.344	
Serum creatinine (mg/dL)	1.0	(0.2)	0.9	(0.2)	0.496	

Abbreviations: ACE, angiotensin converting enzyme; ARB, angiotensin II receptor blockers; eGFR, estimated glomerular filtration rate; NSAID, nonsteroidal anti-inflammatory drug; PPI, proton-pump inhibitor; rhPTH, recombinant human parathyroid hormone; SD, standard deviation.

*denotes *P*-value <0.05.

## Discussion

With the absence of long-term, head-to-head randomized trials for rhPTH(1–84), this is the first study to evaluate the difference in eGFR between chronic HypoPT patients treated with rhPTH(1–84) versus those not treated with rhPTH(1–84) over an extended period. Specifically, the trends in eGFR changes during a 5-year follow-up among rhPTH(1–84)-treated and control patients were compared. After adjusting for potential confounding from cross-cohort differences in patient characteristics, the historical control cohort, who did not receive rhPTH(1–84), exhibited a significant decline in mean eGFR compared with the stable eGFR trend for the rhPTH(1–84) cohort over a 5-year period. This result was supported by a sensitivity analysis using propensity score weighting to compare the difference in eGFR change between more balanced cohorts. The evidence across the analyses in this study suggests that long-term treatment with rhPTH(1–84) may confer benefits in preserving eGFR in patients with chronic HypoPT.

The underlying reason for the estimated differential in eGFR trends by study cohort merits future research, but one potential explanation could be that conventional therapy does not address the pathophysiologic deficit of PTH in HypoPT. Conventional therapy corrects hypocalcemia by increasing intestinal calcium absorption. However, it does not affect the mechanisms for renal calcium reabsorption and urinary phosphate excretion. In fact, it is not uncommon for patients to experience increased urine calcium excretion while simultaneously achieving satisfactory serum calcium levels. Similarly, the serum phosphate concentration and the calcium-phosphate product may also be increased with conventional therapy. It is reported that elevated urinary calcium is associated with an increased risk of nephrocalcinosis and nephrolithiasis and, consequently, may predispose to the development of chronic kidney disease (CKD) (11, 14, 23, 24). This is also supported by a retrospective chart review study reporting significantly higher rates of developing CKD (stage 3–5) than age-appropriate adjusted rates in patients with HypoPT treated with conventional therapy (11). Based on these findings, it seems reasonable to expect that deteriorating eGFR observed in the historical control cohort in this study may be related to uncorrected renal calcium handling with conventional therapy. In contrast, rhPTH(1–84) has been shown to reduce the need for supplemental calcium and reduce the serum phosphate and the serum calcium-phosphate product, which all reduce the renal burden for calcium excretion (25). It has been shown that urinary calcium excretion falls over time with rhPTH(1–84) treatment (26, 27). Reduced urinary calcium excretion may decrease the risk of renal dysfunction in patients with HypoPT, although rigorously controlled clinical trials are required to validate this hypothesis further.

Another challenge of conventional therapy with calcium and active vitamin D is to maintain stable calcium homeostasis. In particular, when patients experience more severe HypoPT symptoms, additional calcium supplements may be required, which may result in fluctuations in serum and urinary calcium levels with an inadequate calcium dose (10). The impact of hypercalcemia and calcium-phosphate homeostasis at the renal level has also been examined in a case-controlled study using the Danish National Patient Registry (28). A higher number of hypercalcemic episodes and a higher calcium-phosphate product were shown to be significantly associated with an increased risk of renal disease in that study. Conversely, treatment with rhPTH(1–84) may help to attenuate fluctuations in serum calcium levels in spite of reduced requirements for supplemental calcium and active vitamin D. Future studies focusing on the long-term results of biochemical monitoring (eg, 24-hour urine calcium, serum calcium, and phosphate levels), along with serum creatinine and eGFR, in patients with chronic HypoPT receiving different therapies would provide further insights into how rhPTH(1–84) may help to preserve eGFR.

The results of this study should be interpreted in light of its limitations, some of which are inherent to all observational studies. First, as is the case for any retrospective study, unmeasured confounding variables could influence the results. The EHR database reflects a real-world setting, and thus, baseline characteristics (eg, etiology of HypoPT) of the comparable control group were necessarily limited. To address this potential issue, several approaches were incorporated in the study design to mitigate cross-population differences. This included the use of similar inclusion/exclusion criteria of the 2 study cohorts, fitting multivariable regression models to account for the differences in baseline characteristics of clinical importance, and conducting a sensitivity analysis using propensity score-weighted populations to further control for potential confounding. A second limitation is that the EHR database may be subject to reporting and coding errors or data omission. For instance, medical history and concomitant medication use of the historical control cohort were identified by diagnosis and GPI/HCPCS drug codes. This approach could reduce the “capture rate” in comparison to the data available from the clinical trials. Furthermore, real-world consumption of the conventional therapies may be underestimated for the control cohort since medical services outside of the Geisinger health system were not reflected in the pharmacy data in the EHR database. Similarly, the longitudinal change in biological monitoring (eg, serum calcium-phosphate product) and patients’ CKD stage could not be evaluated or confirmed in the historical control cohort because laboratory data (eg, albumin creatinine ratio, red and white blood cell counts) were not available. Information on the assays and methods that were used to measure biological parameters (eg, creatinine level) was not available in the EHR database either. Third, the impact of changes in muscle mass on the longitudinal trends of creatinine was not evaluated, as the equation used to estimate GFR in this study (ie, CKD-EPI) accounts for body surface area (BSA) by intrinsic design. While another approach, Cockcroft-Gault equation, although feasible to estimate creatinine clearance without accounting for BSA, also reflects both glomerular and tubular creatinine clearance and may overestimate GFR by up to 40% in younger individuals without CKD (29). The impact of muscle mass on the observed change in eGFR may be modest in this study, as mean body weight remained stable throughout the 5-year follow-up period for both rhPTH(1–84) (baseline vs Year 5: 91.1 kg vs 90.8 kg) and historical control cohorts (83.0 kg vs 83.6 kg). Given these considerations, the CKD-EPI equation was considered an appropriate approach. Fourth, this study was limited by the small sample size, with only 122 individuals being analyzed. However, HypoPT is a rare disease, and this is the largest study to date evaluating changes in eGFR during the long follow-up period. Finally, the control and comparator cohorts were derived from different databases. The rhPTH(1–84) cohort included patients who were enrolled in the RACE or NCT02910466 trial, while the historical control cohort included patients with a Geisinger health plan or who had received care at a Geisinger facility. Nevertheless, the results give important new considerations with regard to changes in eGFR over time in patients with HypoPT treated by conventional means or with rhPTH(1–84).

## Conclusions

Among patients with chronic HypoPT, treatment with rhPTH(1–84) was associated with the preservation of eGFR during a 5-year period, contrasting with a decline in eGFR among patients who were not treated with rhPTH(1–84). Further research with a prospective experimental design is needed to substantiate these findings and to reveal putative underlying mechanisms for how PTH replacement may preserve kidney function in HypoPT.

## Data Availability

The datasets generated during and/or analyzed during the current study are not publicly available but are available from the corresponding author on reasonable request.

## Additional Information

***Disclosure Summary:*** Nicole Sherry is an employee of Shire (a Takeda company). Kristina S. Chen and Alan Krasner were employees of Shire (a Takeda company) during the time of the study’s conduct. Elyse Swallow, Jing Zhao, and Jessie Wang are employees of Analysis Group, Inc., which received payment from Shire (a Takeda company) for contracted research. Elvira O. Gosmanova, Gary C. Curhan, Mishaela Rubin, Markus Ketteler, and John Bilezikian provided research and clinical consultation for Shire (a Takeda company). Elvira O. Gosmanova is an employee of the Department of Veterans Affairs. Opinions expressed in this paper are those of the authors’ and do not necessarily represent the opinion of the Department of Veterans Affairs.
